# Temporal Evolution of Hypertrophic Olivary Degeneration in a Pediatric Patient: A Case Report and Review of Literature

**DOI:** 10.7759/cureus.11109

**Published:** 2020-10-23

**Authors:** Michael Ortiz Torres, Juan C Vicenty-Padilla, Karla C Cay-Martinez, Eduardo J Labat, Juan Vigo-Prieto

**Affiliations:** 1 Neurological Surgery, University of Missouri School of Medicine, Columbia, USA; 2 Neurological Surgery, Brigham and Women's Hospital, Harvard Medical School, Boston, USA; 3 Pediatric Neurology, Columbia University, New York, USA; 4 Neuroradiology, University of Puerto Rico, Medical Sciences Campus, San Juan, PRI; 5 Neurological Surgery, University of Puerto Rico, Medical Sciences Campus, San Juan, PRI

**Keywords:** hypertrophic olivary degeneration, guillain-mollaret triangle, dentato-rubro-olivary tract, pediatric, pilocytic astrocytoma

## Abstract

Hypertrophic olivary degeneration (HOD) is a rare type of neuronal degeneration seen after interruption of the dentato-rubro-olivary tract also known as the Guillain-Mollaret triangle (GMT). It is associated with hypertrophic changes of the inferior olive. Commonly reported in adults, this lesion presents with ataxia and oculopalatal myoclonus. Up to date, few cases have been published in the literature that refer to pediatric cases. This diagnosis is particularly important in the setting of brainstem tumor surgery as it should not be confused for tumor recurrence or metastasis, in turn avoiding unwarranted surgical intervention. We present the case of a 15-year-old male who underwent resection of a left superior cerebellar peduncle (SCP) pilocytic astrocytoma. On follow-up, magnetic resonance imaging (MRI) demonstrated evidence of mild residual tumor as well as progressive engorgement of the inferior olivary nucleus (ION). The patient was clinically asymptomatic and has since been observed expectantly without any issues. We were able to pinpoint the most probable location of injury in our patient’s GMT. HOD remains a somewhat obscure entity. Its presentation may be early and not accompanied by significant neurologic findings, in contrast to what has been previously reported. Particularly in neoplastic cases, it may represent a diagnostic challenge and could be easily confused for tumor recurrence. A multidisciplinary approach for this entity, as with other pathologies, is of particular importance. Its proper recognition will result in the best outcomes for the patient.

## Introduction

The Guillain-Mollaret triangle (GMT), also known as the dentato-rubro-olivary tract, is a synaptic circuit between the ipsilateral red nucleus (RN), the ipsilateral inferior olivary nucleus (ION), and the contralateral dentate nucleus (DN) [1]. As part of the GMT, the RN is connected to the ION via the central tegmental tract (CTT) and to the DN via the superior cerebellar peduncle (SCP), and the DN is connected to the ION via the inferior cerebellar peduncle (ICP). The GMT serves as a connection between the brainstem and the cerebellum and its primary function is modulation of spinal cord motor activity [1,2]. Interruption of the GMT can sometimes result in hypertrophic olivary degeneration (HOD), a rare type of transsynaptic degeneration [3]. HOD was initially described in a post-mortem examination by Oppenheim in 1887 [4] and more extensively studies by Guillain and Mollaret, who described its association to the dentato-rubro-olivary tract in 1931 [5]. HOD is classically associated with a syndrome consisting of ataxia, palatal myoclonus, and ocular myoclonus [6,7], as well as with engorgement of the ION on magnetic resonance imaging (MRI) [8]. HOD represents a diagnostic challenge, particularly in cases involving neoplastic processes, as it may be confused with tumor metastasis or recurrence. The neurosurgical literature concerning HOD is limited, particularly in pediatric patients [9,10].

We present the case of a 15-year-old male who presented with a left SCP pilocytic astrocytoma and underwent resection. On subsequent post-operative imaging, he presented with findings consistent with HOD, but he did not have all of the associated clinical findings.

## Case presentation

**Figure 1:**
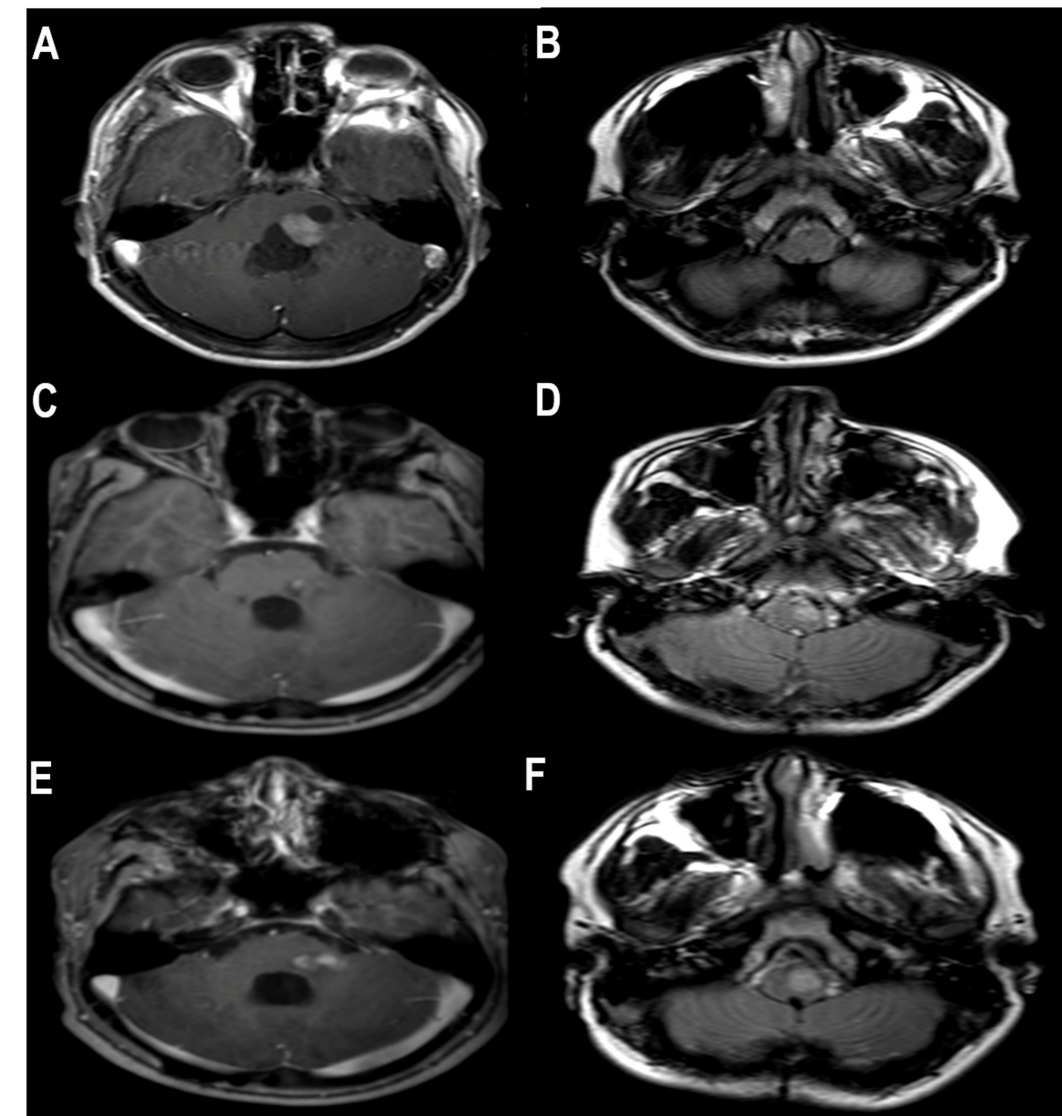
Anatomic-radiologic correlation A: Pre-operative T1 post-contrast MR at the level of the left SCP demonstrating a solid contrast-enhancing lesion at the left SCP with an associated cyst. B: T2 FLAIR at the level of the ION demonstrating no changes in the ION. C: Four-month post-operative T1 post-contrast MR at the level of the left SCP demonstrating mild residual tumor at the left SCP. D: T2 FLAIR at the level of the ION demonstrating hyperintensity and mild hypertrophy of the ION. E: 11-month post-operative T1 post-contrast MR at the level of the left SCP demonstrating interval growth of residual tumor at the left SCP. F: T2 FLAIR at the level of the ION demonstrating increased hyperintensity and marked hypertrophy of the ION. MR= magnetic resonance, SCP= superior cerebellar peduncle, ION= inferior olivary nucleus, FLAIR= fluid-attenuated inversion recovery.

A 15-year-old male without a previous history of systemic illness was evaluated for progressive headaches and discoordination. Physical examination showed left-sided dysmetria, left-sided fast-phase nystagmus, and ataxia. Brain MRI revealed the presence of a left cerebellar peduncle contrast-enhancing lesion with an associated cyst, highly suggestive of a pilocytic astrocytoma (Figure 1A). He underwent surgical resection of the lesion via a telovelar approach without complications. Histopathology confirmed the diagnosis of pilocytic astrocytoma. The patient awoke with a mild left-sided abducens nerve palsy, along with exacerbated dysmetria and ataxia. These findings resolved approximately two weeks later.

Prior to tumor resection, no ION hypertrophy was observed (Figure 1B). On subsequent imaging at four months after surgical intervention, mild residual tumor can be observed (Figure 1C), along with evident T2 fluid-attenuated inversion recovery (FLAIR) hyperintensity and mild hypertrophy of the ION (Figure 1D). Imaging at 11 months post-op revealed mild interval growth of the residual tumor (Figure 1E) and marked ION hypertrophy and T2 FLAIR hyperintensity (Figure 1F). However, at the time of these presentations, the patient was completely clinically asymptomatic. The patient continues routine tumor surveillance at this time.

## Discussion

HOD is an unusual type of neuronal degeneration in that it results in neuronal hypertrophy, as opposed to atrophy, which is the most common type of neuronal degeneration seen in other synaptic circuits [9,11,12]. Multiple factors that affect the dentato-rubro-olivary tract have been identified as causes of HOD, including hemorrhage, neoplasms, trauma, demyelination, inflammation, radiation damage, surgical manipulation, and idiopathic nature [13-19]. The hypertrophy is believed to be the result of disruption of the γ-aminobutyric acid (GABA)-ergic inhibitory effects of the RN over the ION [20], which subsequently leads to neuronal vacuolar necrosis, myelin loss, and gliosis [13]. This phenomenon is of particular importance in neoplastic etiologies of HOD, such as described in our patient, as the new area of hypertrophy should not be confused for a tumoral lesion. The abnormality may be either unilateral or bilateral depending on the location of the lesion [10]. On MRI, HOD is described as a non-contrast enhancing T1 isointense lesion and a T2/FLAIR hyperintense lesion restricted to the ION [8]. These changes can be observed very early in the post-operative course, as in our patient’s case, and may last several years after the initial presentation. Despite being classically associated with a myoclonic syndrome, HOD can be associated with a Holmes tremor or can be asymptomatic as in our case. The pathophysiologic mechanism for these abnormal movements is believed to be secondary to the loss of inhibitory signals - the same mechanism that promotes hypertrophy [20]. Most cases of HOD are managed with observation, given there is no specific treatment developed at this point [11]. Gabapentin has been proposed as a palliative option for the treatment of the ocular nystagmus associated with HOD. Additionally, some authors have suggested surgical intervention for medically refractory cases, such as RN deep brain stimulation, although surgery has not proven to be beneficial in most cases.

**Figure 2:**
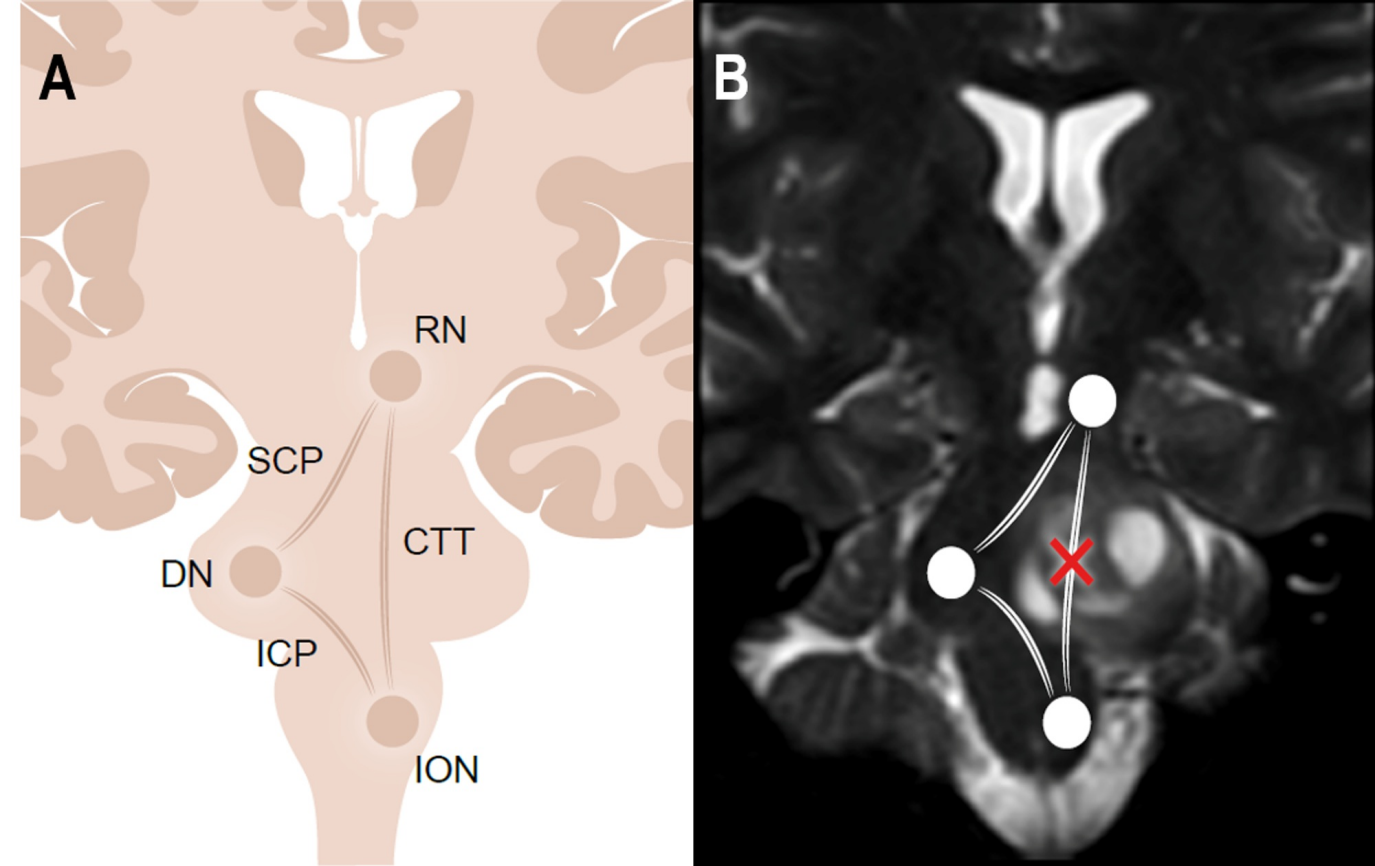
GMT demonstration and application to our patient A: Diagrammatic representation of the GMT. B: GMT superimposed over our patient’s pre-operative coronal T2 MR sequence demonstrating the area of disruption in our patient’s triangle. GMT= Guillain-Mollaret triangle, MR= magnetic resonance, RN= red nucleus, ION= inferior olivary nucleus, DN= dentate nucleus, CTT= central tegmental tract, ICP= inferior cerebellar peduncle, SCP= superior cerebellar peduncle.

We believe the injury to the GMT that caused HOD in our patient was related to the disruption of the CTT during tumor resection (Figure 2). Retrospectively, MRI tractography may have been useful in identifying the CTT pre-operatively although it is unclear whether or not this would have helped prevent GMT injury. Given the tumor had an exophytic component medially, the telovelar approach was the safest route to the lesion. Knowing the position of the CTT may have helped us direct our work away from the tract but would not have necessarily avoided injury.

Familiarity with HOD and the ability to recognize HOD on MR imaging is important, particularly when the surgeon is performing brainstem surgery, given that disruption of the GMT at different areas can result in HOD. The surgeon should not confuse HOD for tumor, as a misdiagnosis may result in unwarranted surgery A multidisciplinary approach, including a conversation with neuroradiology colleagues may provide some additional insight whenever the diagnosis is unclear. If the patient is asymptomatic, observation could be a reasonable option given the behavior of the treated pathology.

## Conclusions

HOD may occur early after brainstem surgery and may not be accompanied by significant neurologic findings. In neoplastic cases, HOD may represent a diagnostic challenge since it could be easily confused for tumor recurrence. A multidisciplinary should result in the best outcomes for the patient.
